# Unraveling the dynamic importance of county-level features in trajectory of COVID-19

**DOI:** 10.1038/s41598-021-92634-w

**Published:** 2021-06-22

**Authors:** Qingchun Li, Yang Yang, Wanqiu Wang, Sanghyeon Lee, Xin Xiao, Xinyu Gao, Bora Oztekin, Chao Fan, Ali Mostafavi

**Affiliations:** 1 Zachry Department of Civil and Environmental Engineering, Texas A&M University, 199 Spence St., College Station, TX 77843 USA; 2 Department of Computer Science and Engineering, Texas A&M University, 199 Spence St., College Station, TX 77843 USA

**Keywords:** Mathematics and computing, Computational science

## Abstract

The objective of this study was to investigate the importance of multiple county-level features in the trajectory of COVID-19. We examined feature importance across 2787 counties in the United States using data-driven machine learning models. Existing mathematical models of disease spread usually focused on the case prediction with different infection rates without incorporating multiple heterogeneous features that could impact the spatial and temporal trajectory of COVID-19. Recognizing this, we trained a data-driven model using 23 features representing six key influencing factors affecting the pandemic spread: social demographics of counties, population activities, mobility within the counties, movement across counties, disease attributes, and social network structure. Also, we categorized counties into multiple groups according to their population densities, and we divided the trajectory of COVID-19 into three stages: the outbreak stage, the social distancing stage, and the reopening stage. The study aimed to answer two research questions: (1) The extent to which the importance of heterogeneous features evolved at different stages; (2) The extent to which the importance of heterogeneous features varied across counties with different characteristics. We fitted a set of random forest models to determine weekly feature importance. The results showed that: (1) Social demographic features, such as gross domestic product, population density, and minority status maintained high-importance features throughout stages of COVID-19 across 2787 studied counties; (2) Within-county mobility features had the highest importance in counties with higher population densities; (3) The feature reflecting the social network structure (Facebook, social connectedness index), had higher importance for counties with higher population densities. The results showed that the data-driven machine learning models could provide important insights to inform policymakers regarding feature importance for counties with various population densities and at different stages of a pandemic life cycle.

## Introduction

COVID-19 has inflicted great loss in both economic and social dimensions. To inform pandemic situations, several studies developed mathematic models to predict the outbreak and trajectory of pandemics to provide important insights for use by policymakers to develop pandemic control measures. For example, Tizzoni et al.^1^ predicted the epidemic spread among three European countries using a susceptible-infectious-recovered (SIR) model that accounted for human mobility. Balcan et al.^2^ used the mathematical model to study global pandemic dynamics considering long-range and short-range mobility patterns. Ferguson et al.^3^ developed the transmission model to test the effects of epidemic control measures (e.g., social distancing) with different virus reproduction rates. Wang et al.^4^ developed a deep learning model to predict epidemic transmission using within-season and between-season observations as features. For the COVID-19 pandemic, many studies developed mathematical models to evaluate containment measures and to predict the potential outbreak and trajectory of COVID-19. Anastassopoulou et al.^5^ predicted the evolution of the spread of COVID-19 based on a susceptible-infectious-recovered-deceased (SIRD) model and the estimation of the reproduction number (R_0_). Block et al.^6^ simulated community spread scenarios of COVID-19 based on social network structures. Chang et al.^7^ integrated the standard susceptible-exposed-infectious-recovered (SEIR) model with the origin–destination mobility networks to fit the trajectory of COVID-19 and predicted infections in the reopening stage. Gatto et al.^8^ also used the standard SEIR model to evaluate the effectiveness of COVID-19 containment measures (such as mobility reduction and social distancing). Cintia et al.^9^ used a regression model to investigate the relationship between human mobility and the transmission of COVID-19 in Italy. Perc et al.^10^ used a simple iteration that relied only on confirmed cases to forecast the spread of COVID-19 in the United States, Slovenia, Iran, and Germany. Petropoulos and Makridakis^11^ implemented a simple time-series forecasting to predict the spread of COVID-19 based on the data of confirmed cases, deaths and recoveries. Tomar and Gupta^12^ used the LSTM model to predict the spread of COVID-19 in India and discussed the effectiveness of pandemic control measures, such as social distancing and lockdown. Chimmula and Zhang^13^ also developed long short-term memory (LSTM) deep learning model to predict the transmission of COVID-19 in Canada. The existing mathematical models seek to predict the trajectory of epidemics/pandemics based on a limited number of features, such as mobility patterns, reproduction rates of virus, observations within and between seasons, number of confirmed cases, deaths and recoveries. Most of the existing mathematical models, however, could account for only a limited number of features and could not simultaneously examine the importance of heterogeneous features, such as social demographics, population activities, mobility patterns, disease-related attributes, and social network structure-based various datasets.

Various studies related to COVID-19 have highlighted multiple influencing factors that would affect the pandemic spread. Dowd et al.^14^ highlighted the importance of social and demographics attributes (mainly focusing on age structures of populations) affecting infection rates in populations. Nepomuceno et al.^15^ found that other demographic factors could affect the spread of COVID-19. Multiple studies have reported the effects of population density^16–18^, household size and composition, hygienic and sanitary conditions, access to healthcare services, case notification systems, and economic disparities^19^ on the trajectory of the COVID-19 infections. Yancy^20^, Dyer^21^, Laurencin^22^, and Millett et al.^23^ pointed out the racial and ethnic disparities of COVID-19 that hit minorities harder. In addition to the social and demographic factors, additional studies have reported the role of population activities, such as visits to points of interests (e.g., hospitals, restaurants and recreation centers)^24–26^ and staying at home^27–29^ as they affect transmission risks of COVID-19. Kraemer et al.^30^, Badr et al.^31^, Jia et al.^32^, Linka et al.^33^, Hâncean et al.^34^and Askitas et al.^35^ investigated the extent to which human mobility would affect the spread of COVID-19. Liu et al.^36^, Zhang et al.^37^, You et al.^38^, and Shim et al.^39^ examined the effects of disease attributes, such as the reproduction number, R_0_, on the trajectory of COVID-19. Furthermore, Bucur^40^, Block et al.^6^, and Kuchler et al.^41^ discussed how the social network structures would affect the spread of COVID-19 in communities. A recent deep learning model proposed by Ramchandani et al.^42^ accounted for several heterogeneous features (e.g., social demographics, population activities, and mobility pattern). While the existing studies inform about various heterogenous features affecting the trajectory of COVID-19 spread, limited knowledge exists about the importance of these features across different cities and communities and at different stages of the pandemic spread. For example, mobility restriction orders may have greater effect on counties with higher population densities^43^, and long-distance mobility restriction orders are more effective in the outbreak stage of the epidemic, while local control measures, such as shelter-in-place and social distancing orders, would be more effective after the outbreak stage^30^. Unraveling the importance of various features in the trajectory of pandemics is a critical element for predictive surveillance and data-driven policy formulation. Hence, to address this important knowledge gap, we aim to answer two research questions in this paper: (1) The extent to which the importance of heterogeneous features evolved at different stages (e.g., outbreak stage, social distancing stage, and reopening stage); (2) The extent to which the importance of heterogeneous features varied across counties with different characteristics.

To answer these two research questions, we examined 23 features related to social demographics, population activities, mobility, social network structure, and disease-related attributes. We collected features of 2787 counties from March 24 to June 23, 2020, the COVID-19 pandemic. We divided the spread timeline of COVID-19 into three stages: the outbreak stage, the social distancing stage, and the reopening stage, according to the non-pharmacological interventions issued by state and local governments in the U.S. After the initial outbreak was identified and the number of COVID-19 cases in the U.S. surged, most counties issued social distancing and shelter-in-place orders. Human mobility (e.g., within county commute and across county travels) and population activities (e.g., point of interest visits) were greatly reduced in this social distancing period. After the confirmed cases of COVID-19 was reduced, counties lifted social distancing and shelter-in-place orders. Human mobility and population activities gradually recovered in this reopening period. Existing studies showed that temporal features may greatly vary at these different stages and could affect the spread of COVID-19^32,44^. Therefore, dividing the timeline of COVID-19 spread into these three stages would provide more insights into dynamic feature importance evaluation, providing insights regarding relevant intervention policies and containment strategies. Table 1 provides a summary of the examined features and their underlying data. (Refer to the supplemental materials for the elaboration on each feature and data source.) Based on these features, we built a set of data-driven random forest models to study the dynamics of importance of each feature during different stages of COVID-19, and we evaluated the importance across counties with different population densities. We would like to note that the purpose of this paper is not to build a state-of-the-art prediction model of COVID-19, although the data-driven model could be a complementary tool to prediction models.

**Table 1:** Collected features for the data-driven model.

Social demographic features	Population density	Constant feature
	Gross domestic product (GDP)	Constant feature
	Socioeconomic status	Constant feature
	Household composition and disability	Constant feature
	Minority status and language	Constant feature
	Housing type and transportation	Constant feature
	Epidemiologic factors	Constant feature
	Healthcare system factors	Constant feature
	Overall COVID-19 community Vulnerability index (CCVI)	Constant feature
Population activity features	Point-of-interest visits	Time-dependent feature
	Social distancing index (SDI)	Time-dependent feature
	Urban activity index (social)	Time-dependent feature
	Urban activity index (work)	Time-dependent feature
	Urban activity index (traffic)	Time-dependent feature
	Urban activity index (home)	Time-dependent feature
	Venables Distance	Time-dependent feature
Within-county mobility features	Cuebiq county mobility index (CMI)	Time-dependent feature
	Cuebiq shelter-in-place index (SIP)	Time-dependent feature
Across-county mobility features	County in-degree centrality	Time-dependent feature
	County out-degree centrality	Time-dependent feature
	Colocation degree centrality	Time-dependent feature
Disease attribute feature	Reproduction number (R_0_)	Time-dependent feature
Social network structure feature	Social connectedness index	Constant feature

## Methodology

**Figure 1:**
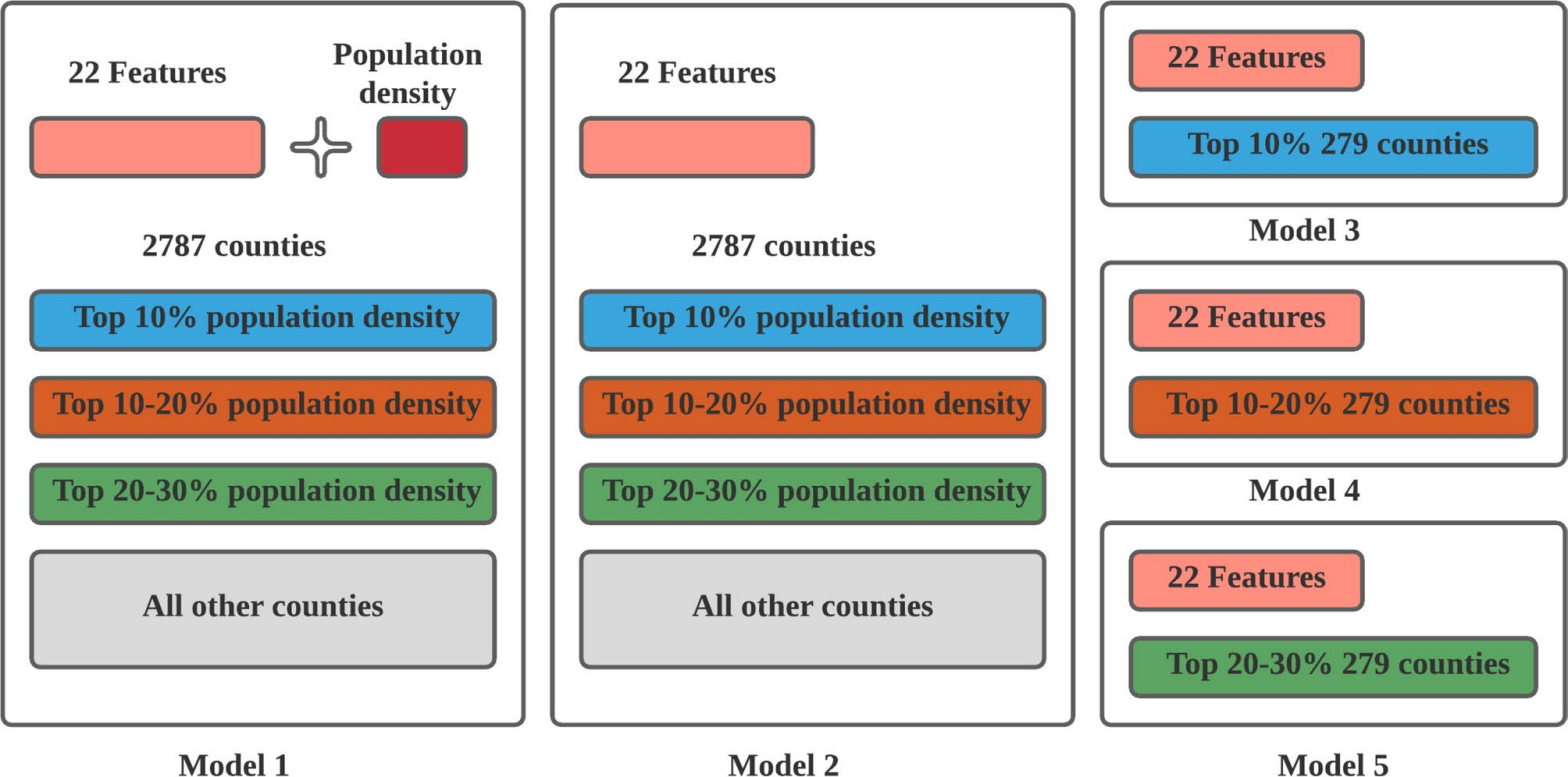
Features and counties included in five random forest models. Model 1 includes all counties and collected features. Model 2 includes all counties and features excluding population densities. Models 3, 4 and 5 include all features excluding population densities and counties with top 10%, 10–20%, and 20–30% population densities respectively.

**Figure 2:**
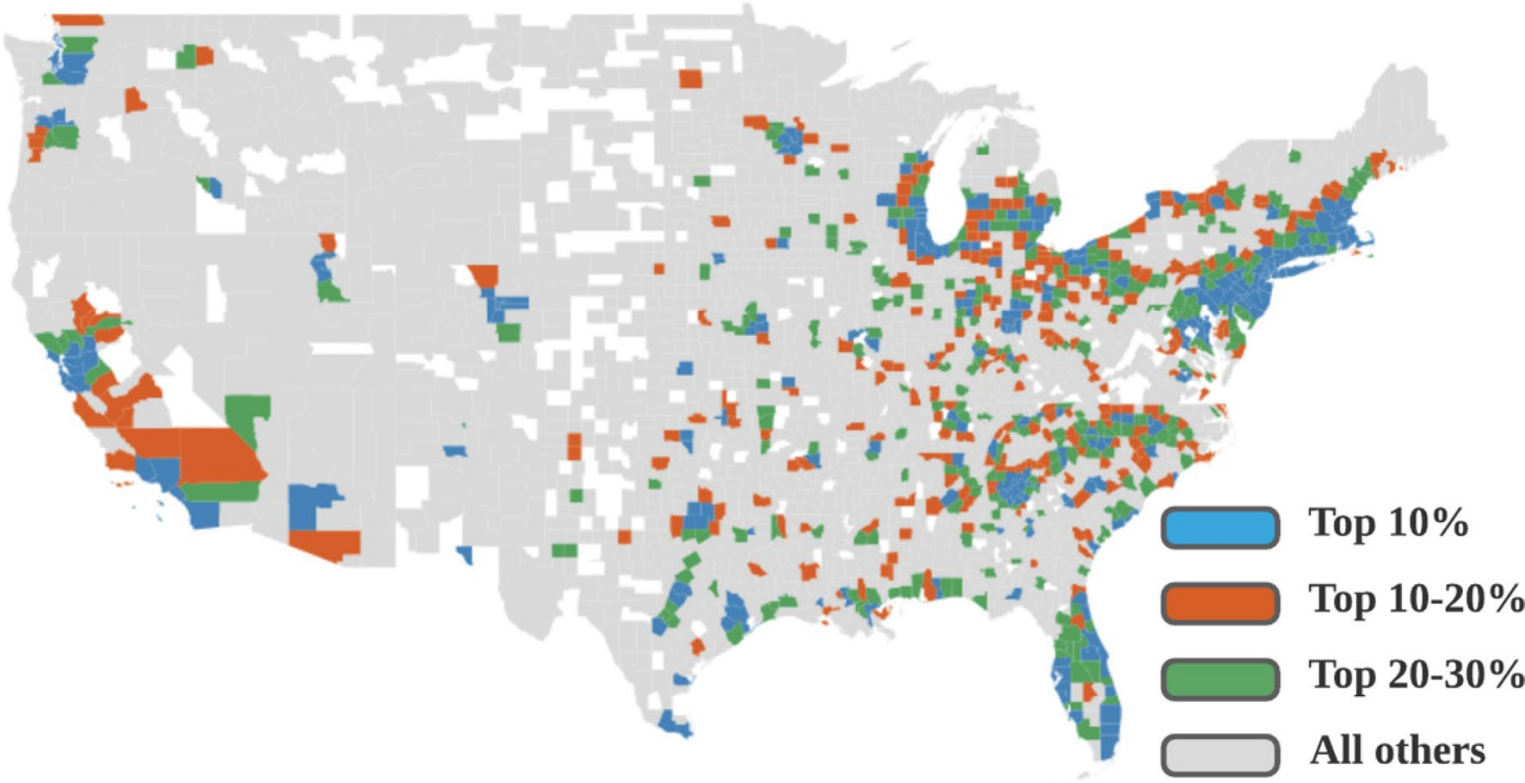
Spatial visualization of studied 2,787 counties with different population densities. Blue: counties with top 10% population density. Orange: counties with top 10–20% population densities. Green: counties with 20–30% population densities. Gray: all other counties.

For each week from March 24, 2020, to June 23, 2020, we created five random forest classifier models with nested cross validation using 23 county-level features. Each model was trained and tested using ten-fold cross validation and each training fold was further divided into five-fold training and validation folds to tune the model hyper-parameters. Each model was trained and tested for each week in the studied period to investigate the evolution of feature importance during the COVID-19 pandemic. Different random forest models tend to divide counties into clusters based on population densities. Population density is shown to be a dominant feature affecting the number of infected cases. To examine the effects of features other than population density, we created models for clusters of counties with varying population densities. Figure 1 illustrates included features and counties in five sets of random forest models. Figure 2 illustrates clusters of counties with varying population densities in models. We explain each random forest model in the following sub-sections.

### Two base models

We built two base random forest models. Model 1 includes all 23 features as independent variables; model 2 excludes population density, to enable assessment of the effect of other features in the absence of population density. The base models include 2787 counties in the United States. The dependent variable in each base model is composed of five classifications of the weekly new confirmed cases per 100,000 population (CPP) for each county provided by Centers for Disease Control and Prevention and Johns Hopkins University. For each week, we put counties with zero CPP in one classification. Then, we evenly divided the rest of counties into four classifications based on quantiles of CPP. Figure S1 in the supplementry materials illustrates histograms of the five classifications in each week. We used these two models as the baseline to compare feature importance in other models.

### Models of counties with different population densities

This set of random forest models include models 3, 4 and 5. Each model comprises 279 counties with top 10 (model 3), top 10 to top 20 (model 4), and top 20 to top 30 (model 5) percent of population densities among the 2787 counties. The dependent variable in these models is the same as that in the base models (i.e., five classifications based on the CPP in each week). The independent variables in this set of models include 22 features, excluding population density of each county. We used this set of models to examine feature importance of counties across different population density clusters.

We examined the results of feature importance of each weekly model. Random forest modeling uses aggregated decreases in Gini importance of features to determine feature importance based on Eqs. (1) through (5)^45,46^. A greater aggregated decrease in a feature implies that the feature is more important.

1 $$\Delta G\left( j \right) = ~G\left( j \right) - \frac{{|L_{j} |}}{{\left| j \right|}}G\left( {L_{j} } \right) - \frac{{|R_{j} |}}{{\left| j \right|}}G\left( {R_{j} } \right)$$

where $G(j)$ is the Gini importance calculated accord to Eq. (2), and *j* is the partition at node *j*, while $L_{j}$ and $R_{j}$ are the left and right child nodes of partition *j*, respectively.

2 $$G\left( j \right) = ~\mathop \sum \limits_{{i = 1}}^{C} p_{i} \left( {1 - p_{i} } \right)$$

where *C* is the total number of classes while $p_{i}$ is the probability of a datapoint from *j* in class *i*.

3 $$F_{i} = ~\mathop \sum \limits_{{j:node\;j\;splits\;on\;feature\;i}} \Delta G\left( j \right)$$

4 $$Norm\;F_{i} = ~\frac{{F_{i} }}{{\mathop \sum \nolimits_{{j \in all\;features\;in\;one\;tree}} F_{j} }}$$

where $F_{i}$ is the importance of feature *i* in one decision tree and is normalized between 0 to 1 according to Eq. (4). Then final feature importance in the forest is determined by averaging normalized feature importance of all the trees (Eq. 5).

5 $$RF\;F_{i} = ~\frac{{\mathop \sum \nolimits_{{j:all\;trees}} Norm\;F_{{ij}} }}{T}$$

where *T* is the total number of trees in the forest and $Norm\;F_{{ij}}$ is the normalized importance of feature *i* in tree *j*.

Note that previous studies showed that feature importance based on the reduction of Gini importance would perform worse for categorical features, and some studies proposed new algorithms to correct the bias^47–49^. In this paper, we still used the reduction of Gini importance because the features in our models are all numerical features.

## Results

**Figure 3:**
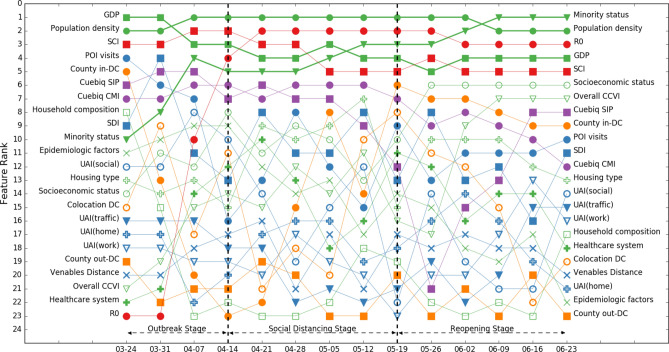
Rank of feature importance of model 1 throughout three stages of COVID-19. Color coding of 23 features: green: social demographic features, blue: population activity features, purple: within-county mobility features, orange: across-mobility features, and red: disease attribute feature and social network structure feature. The legend also distinguishes each feature. Features with greater importance are highlighted with thicker lines. Population density shows high importance throughout three stages of COVID-19.

**Figure 4:**
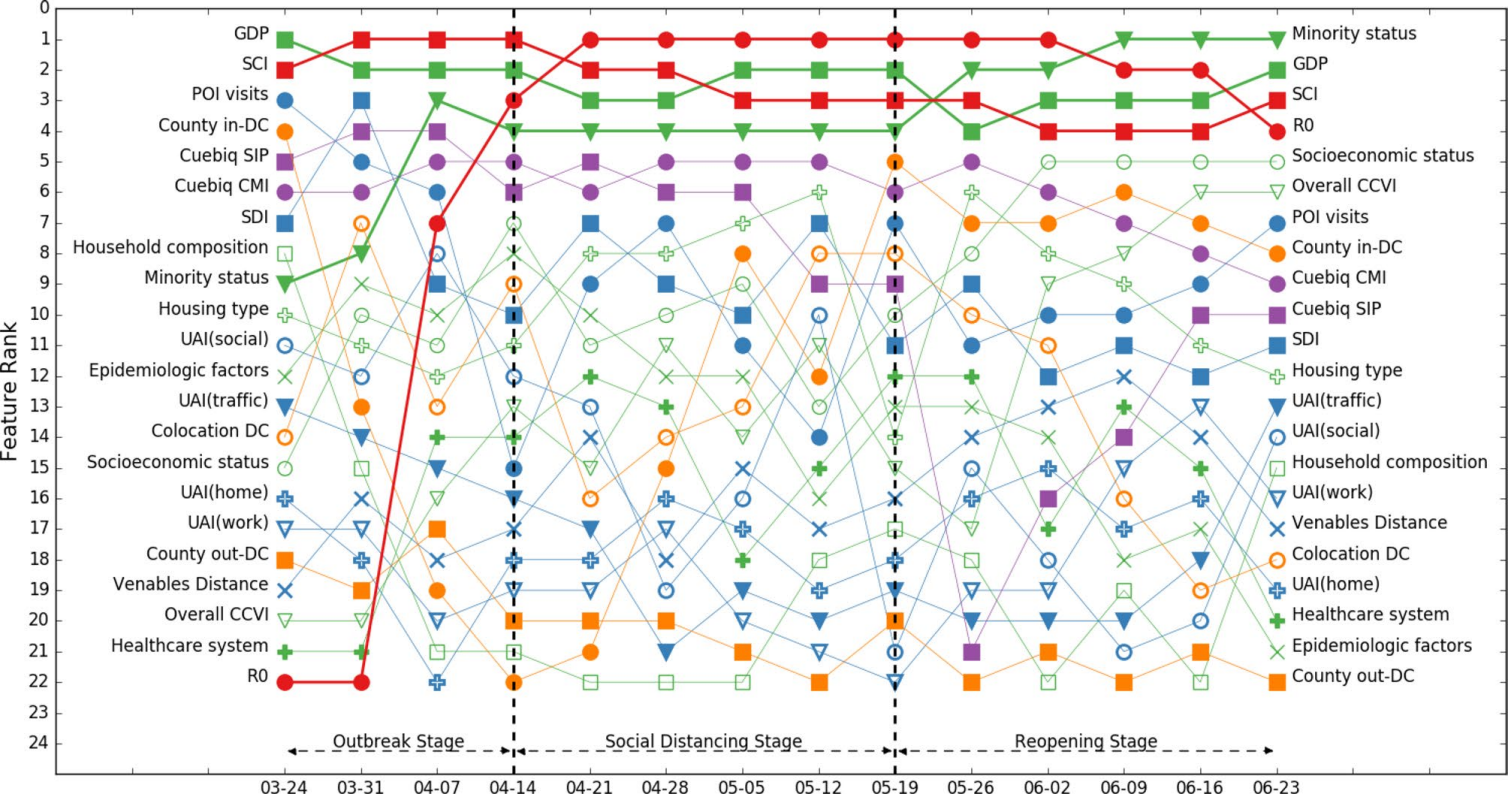
Rank of feature importance of model 2 throughout three stages of COVID-19. Social demographic features and social network structure features show high importance throughout three stages of COVID-19.

We found disparate patterns of feature importance at different stages of the COVID-19 pandemic and across counties with different population densities. Figures 3 and 4 illustrate the ranks of feature importance in model 1 and model 2, which include all 2787 counties (Refer to the supplementary materials for information about the accuracy of the random forest models).

### Feature importance in models 3 and 4

We can observe from Figs. 3 and 4 that ranks of feature importance of models 1 and 2 are quite close. Some features retained high importance while some features showed varied importance in different stages of the COVID-19 pandemic.

A. Features keeping high importance across stages

We can find that gross domestic product (GDP), population density, and social connectedness index (SCI) maintained high feature importance across all stages of COVID-19 in models 1 and 2, both of which include all 2787 counties. Population density stayed within the top two most important features, while GDP and SCI stayed in the top 5 most important features. Although we removed the influence of population size in the weekly confirmed cases—we considered confirmed cases per 100,000 population—the results of feature importance still indicate that population density has a significant impact on CPP. This is consistent with the results of existing studies that examined the effects of population density on the spread of COVID-19^16,17^. GDP is another important social demographic variable. GDP would highly affect other social demographic variables, such as household size and composition, hygienic and sanitary conditions, access to healthcare services, case notification systems, and economic disparities. Existing research has highlighted the importance of these social demographic variables in the spread of the COVID-19 pandemic^15^. Furthermore, SCI captures the effects of social networks; the result indicates that social networks greatly affect the risks of virus spread during all stages of the pandemic. This result is consistent with existing studies that more connected social network structures are at a greater risk of virus spread during the pandemic^6,40,41^.

B. Features showing increasing importance across stages

We can observe that the importance of reproduction number (R_0_), minority status, socioeconomic status, and COVID-19 community vulnerability index (CCVI), all of which showed increasing importance across stages.

R_0_ was of low importance in the outbreak stage and spiked to the highest importance during the social distancing stage and remained in the top four most important features in the reopening stage. This result implies that R_0_ is an important feature in determining the extent of disease spread when community spread begins. The importance of minority status showed a similar pattern: it was of relatively low importance in the outbreak stage, then rose to the top four in the social distancing stage and became the highest-importance feature in the reopening stage. This result supports the findings of other studies by Yancy^20^, Dyer^21^, Laurencin^22^, and Millett et al.^23^ that reported a greater exposure to the virus in racial minority populations. Another two social demographic features, socioeconomic status and CCVI, also showed relatively low importance in the outbreak stage, increasing importance in the social distancing stage, and relatively high importance in the reopening stage. The results not only highlight the importance of social demographic features in the spread of virus, but also shed light on the criticality of incorporating a functional timeline that takes into account relevant features when developing pandemic control policies. For example, reopening planning should account for populations with different socioeconomic status and CCVI because these two features demonstrate high importance in the reopening stage.

C. Features showing decreasing importance across stages

We found that the importance of two within-county mobility features, Cuebiq county mobility index (CMI) and Cuebiq shelter-in-place index (SIP) showed an overall decreasing trend across stages in two base models. The importance of both was high in the outbreak stage. This result implies the importance of mobility reduction measures in the initial outbreak to slow down community spread^30^. The importance of CMI was also high in the social distancing stage and showed a decreasing trend in the reopening stage, while the importance of SIP showed a decreasing trend in the social distancing stage and became increasingly high in the reopening stage. These results could imply that counties which maintained their social distancing practices even after reopening were more likely to maintain or to decrease their number of infection cases.

D. Other highlighted features

For features related to population activities, point-of-interest (POI) visits had high feature importance at the beginning of the outbreak stage, but its importance started waned in the following weeks, reaching lowest importance at the end of the outbreak stage. In the social distancing stage, the importance of POI visits fluctuated: it increased during the first two weeks then decreased in the following two weeks. In the reopening stage, the importance of POI visits showed an increasing trend. The result could imply the importance of maintaining the reduction in POIs visits in the social distancing and reopening stage for the containment of the virus spread. The social distancing index (SDI), although showing a fluctuating pattern, had a relatively high feature importance across all three stages. Similarly, this result supports the importance of voluntary and mandatory social distancing measures for virus spread reduction across all stages.

The importance of cross-county mobility features and county out-degree and in-degree centrality revealed important insights regarding travel reduction. County in-degree centrality, like POI visits, had relatively high importance at the beginning of the outbreak stage but showed a decreasing trend until the end of outbreak stage. In the social distancing stage, the importance of county in-degree centrality showed an increasing trend, reached its highest importance rank at the end of social distancing stage and kept relatively high feature importance in the reopening stage. The importance of county out-degree centrality, on the other hand, kept low across three stages of COVID-19. The results highlight the importance of travel reduction and limited cross-county movements in all stages of the pandemic, especially for the movements into counties. Counties should limit and monitor the number of travelers from other counties to effectively contain the spread of the virus from their counties.

### Feature importance across population density clusters

**Figure 5:**
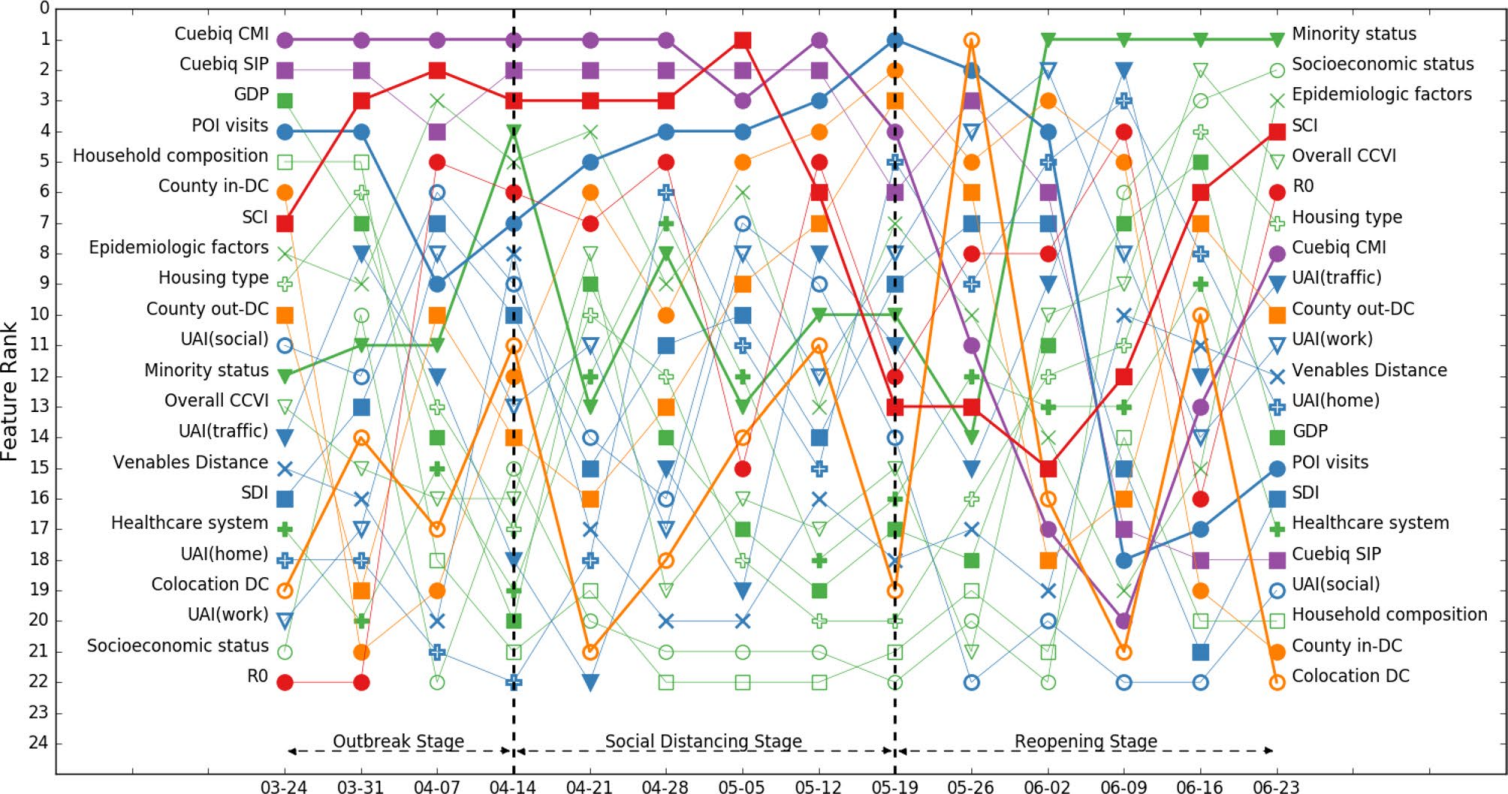
Rank of feature importance of model 3 throughout three stages of COVID-19. Mobility and social network structure features show high importance in counties with high population densities.

**Figure 6:**
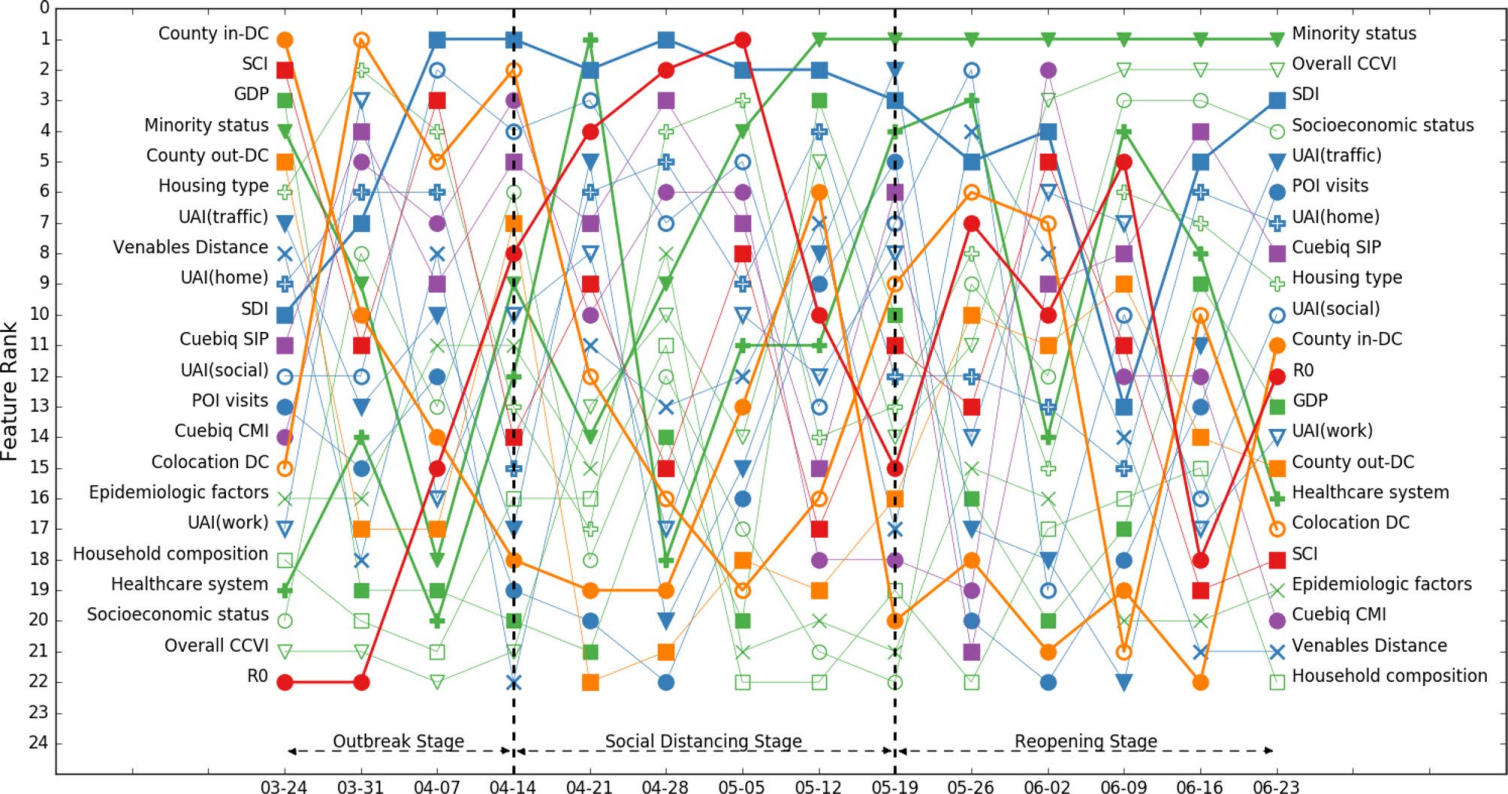
Rank of feature importance of model 4 throughout three stages of COVID-19. Social distance index shows high importance in counties with low population densities.

**Figure 7:**
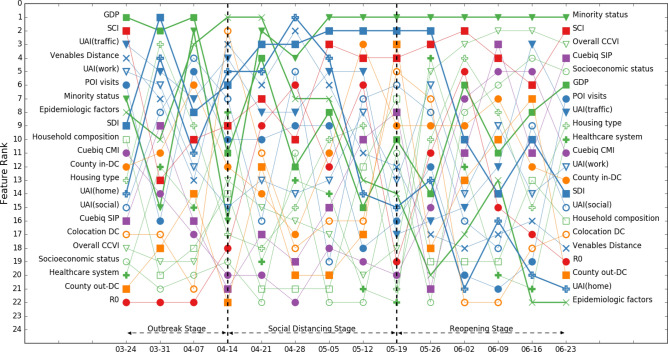
Rank of feature importance of model 5 throughout three stages of COVID-19. Social distance index shows high importance in counties with low population densities. Social demographic features have high importance in counties with low population densities.

In the next step, we investigated feature importance for counties with different population densities. Figures 5, 6 and 7 illustrate models 3, 4 and 5, which encompass data from 279 counties with the top 10%, the top 10–20%, and top 20–30% population densities among the 2787 counties.

We can observe from Figs. 5, 6 and 7 that feature importance shows different patterns across population density clusters. Some features showed high importance only in the clusters with high population density, and some features showed lower importance in population density clusters compared with importance of the two base models.

A. Features having high importance in the high-population density cluster

We can observe that two within-county mobility features, Cuebiq CMI and SIP, showed high importance only in the top 10% population density cluster (model 3). For counties with lower population densities in models 4 and 5, the importance of these two within-county mobility features decreased, with the lowest importance in model 5. In model 3, these two features showed high importance in the outbreak and social distancing stages. In the reopening stage, the importance of SIP showed a decreasing trend, while CMI decreased in the first three weeks then showed an increasing trend again. The results are consistent with existing studies that indicate that mobility restriction orders could be more effective in counties with higher population densities^43^. Also, mobility restriction orders are more effective in early stage (i.e., outbreak stage) of the epidemic, and local control measures, such as shelter-in-place and social distancing orders, would be more effective after the outbreak stage^30^.

Also, another dominant feature in the base models, social connectedness index (SCI), had a pattern similar to that of Cuebiq CMI. In model 3, SCI showed high importance in the outbreak stage, and the importance started to decrease in the social distancing stage. In the reopening stage, the importance of SCI showed an increasing trend. The results imply that SCI is more important in the county clusters with higher population densities, and SCI had higher and increased importance when there were more and increased human interactions. Hence, policy makers should account for social connectedness in the area with higher population densities.

B. Features showing high importance in low population density clusters

We found that social distance index had higher importance in low population density clusters. SDI represents the portion of the number of digital devices at home divided by the total number of digital devices in the area. In models 4 and 5, SDI showed higher importance in the social distancing stage. In model 3, although SDI had lower importance compared with models 4 and 5, it still showed an increasing importance in the social distance stage. The results could imply that SDI is an important feature for effective pandemic control in the social distancing stage. Also, social distancing may more effectively help pandemic control in counties with low population densities in the social distancing stage. The results show that it is important to develop and monitor social distancing measures, especially for the counties with low population densities.

C. Features showing lower importance and features keeping high importance in various population density clusters

We found that in models 3 through 5, GDP and R_0_ did not show the same importance compared with the base models inclusive of all counties. This result may imply the correlation between GDP, R_0_, and population density. The minority status feature, however, still showed high importance in models of population density clusters, especially in the social distancing and reopening stages. The results indicate that for the county clusters with close population densities, GDP and R_0_ are not important features in examining the spread of virus. Policy makers should pay attention to racial minority groups, especially in the social distancing and reopening stage. Formulating policies that could help racial minority groups may effectively help with the overall pandemic controls.

## Discussion

The majority of the existing literature on epidemic spread modeling and COVID-19 pandemic primarily focuses on standard epidemiological models for examining the effects of population features on disease spread. However, the ability of these models to examine the relative importance of various features across different stages of the disease spread based on various datasets is rather limited. To address this gap, in this paper, we investigated the importance of collected 23 heterogeneous features in the trajectory of COVID-19 using a data-driven machine-learning model comprising 2787 counties in the United States. The results demonstrate the dynamics of feature importance among counties in United States and across three stages of the COVID-19 pandemic. In the models including all 2787 counties, (1) social demographic features, such as GDP and population density, and the feature reflecting social interaction strength, social connectedness index, kept high importance through stages of the COVID-19 pandemics; (2) a virus attribute feature, reproduction number (R_0_), and some social demographic features, including minority status, socioeconomic status, and COVID-19 community vulnerability index (CCVI) showed increased importance in the trajectory of the COVID-19 pandemic; (3) within-county mobility features, Cuebiq county mobility index (CMI) and shelter-in-place (SIP), showed decreased importance across different stages; while in the models with different population densities, the level of importance varied; (4) within-county mobility features showed higher importance in county clusters with higher population densities; (5) GDP and R_0_ did not show the same importance within the models encompassing 2787 counties, while the minority status feature still showed an initial low level and increasing importance across stages; and (6) social distance index (SDI) showed higher importance in county clusters with lower population densities and higher importance in the social distancing and reopening stages.

The results showed consistency with findings of other studies. For example, an action plan for pan-European defence against new COVID-19 variants highlighted the restriction of mobility across borders at the beginning and protection of the vulnerable^50^. Nepomuceno et al.^15^ highlighted that social demographic factors would greatly affect the spread of COVID-19. Also, Yancy^20^, Dyer^21^, Laurencin^22^, and Millett et al.^23^ argued that minority groups are disproportionately affected by COVID-19. Kuchler et al.^41^ showed social network structure would affect the spread of COVID-19 among counties. Furthermore, the results showed that within-county mobility features had higher importance in the model of counties with higher population densities, which is consistent with the argument that mobility restriction orders may have greater effect on counties with higher population densities^43^. Our study, however, examined the importance of multiple heterogeneous features related to population activities, sociodemographic attributes, virus features, and mobility simultaneously. For example, our study revealed that the importance of social network structure decreased in the model of counties with lower population densities. Social distancing index had higher importance in the model of counties with lower population densities. Minority status kept high importance in different population density clusters.

The results could help policymakers develop pandemic control measures and strategies at different levels and at different timepoints. For example, when policymakers develop pandemic control measures at the country level, GDP, population densities, and SCI could be effective indictors to separate counties for customized pandemic control measures. When considering a specific county, such as New York County (also known as Manhattan) that has the highest population density, pandemic control measures could focus on within-county mobility and social network structure in the outbreak and social distancing stages, while minority status, socioeconomic status, and overall COVID-19 community vulnerability index could be employed in the social distancing and reopening stages. In smaller counties (such as Brazos County, Texas), pandemic control measures may need to focus on social distancing in the outbreak and social distancing stages, while in the reopening stage, social demographic factors, such as minority status and CCVI should be accounted for. The feature importance analysis could also suggest what feature indicators should be monitored by public officials in different counties and across different stages of a pandemic.

We would like to note some limitations in this study. We considered only first-order feature importance in this paper. Features may have interactions that deviate from an additive linear explanation at different timepoints and for models of counties with different characteristics. The dynamics of feature interaction in the trajectory of pandemics could be explored in future studies. The results of feature interaction could inform which feature will strengthen or weaken another feature. In this paper, we collected only 23 county level features. Future studies could explore and use more county-level features to complement these results.

## Supplementary Information

Supplementary Information.
