# Supplementary material for: Unraveling the dynamic importance of county-level features in trajectory of COVID-19
Source: Sci Rep. 2021 Jun 22;11:13058. doi: 10.1038/s41598-021-92634-w (PMC8219723; doi:10.1038/s41598-021-92634-w)
Supplement: Supplementary file 1 — Supplementary Information. [file 41598_2021_92634_MOESM1_ESM.docx]

**Unraveling the Dynamic Importance of County-level Features in Trajectory of COVID-19**

# Qingchun Li1*, Yang Yang2, ﻿ Wanqiu Wang3, Sanghyeon Lee4, Xin Xiao5, Xinyu Gao6, Bora Oztekin7, Chao Fan8, Ali Mostafavi9

^1^ Ph.D. student, Zachry Department of Civil and Environmental Engineering, Texas A&M University, 199 Spence St., College Station, TX 77840; e-mail: [qingchunlea@tamu.edu](mailto:qingchunlea@tamu.edu)

^2^ Master student, Department of Computer Science and Engineering, Texas A&M University, 199 Spence St., College Station, TX 77843-3112; e-mail: [yangyangsandy@tamu.edu](mailto:yangyangsandy@tamu.edu)

^3^ Master student, Department of Computer Science and Engineering, Texas A&M University, 199 Spence St., College Station, TX 77843-3112; e-mail: [wanqiu.wang@tamu.edu](mailto:wanqiu.wang@tamu.edu)

^4^ Master student, Department of Computer Science and Engineering, Texas A&M University, 199 Spence St., College Station, TX 77843-3112; e-mail: <mailto:sanghyeonlee@tamu.edu>,

^5^ Master student, Department of Computer Science and Engineering, Texas A&M University, 199 Spence St., College Station, TX 77843-3112; e-mail: [xyx56@tamu.edu](mailto:xyx56@tamu.edu),

^6^ Master student, Department of Computer Science and Engineering, Texas A&M University, 199 Spence St., College Station, TX 77843-3112; e-mail: [xy.gao@tamu.edu](mailto:xy.gao@tamu.edu),

^7^ Master student, Department of Computer Science and Engineering, Texas A&M University, 199 Spence St., College Station, TX 77843-3112; e-mail: [bora@tamu.edu](mailto:bora@tamu.edu)

^8^ Ph.D. Candidate, Department of Computer Science and Engineering, Texas A&M University, 199 Spence St., College Station, TX 77843-3112; e-mail: <mailto:chfan@tamu.edu>,

^9^ Associate Professor, Zachry Department of Civil and Environmental Engineering, Texas A&M University, 199 Spence St., College Station, TX 77840; e-mail: [amostafavi@civil.tamu.edu](mailto:amostafavi@civil.tamu.edu)

**Supplementary Materials**

**1. Collected features for the data-driven random forest model**

***Features related to social demographics:***

**Population density**

We calculated population density of each county (population/square miles), as previous works showed that population density is an important factor influencing the spread of an epidemic ^1,2^. Population density data was calculated based on the county-level Social Vulnerability Index 2018 of United States published by Centers for Disease Control and Prevention (CDC) ^3^.

**Gross domestic product (GDP)**

We included GDP of each county in 2018 as a feature for the model, as previous works showed that GDP could be a vulnerability index for COVID-19. Counties with higher GDP usually have a more robust economy and better health systems compared with counties with lower GDP ^4,5^. We used the 2018 county-level GDP published by United States Department of Commerce ^6^.

**COVID-19 Community Vulnerability Index**

This study incorporates the county-level COVID-19 Community Vulnerability Index developed by Surgo Foundation based on the CDC data ^7^ which comprises seven social and demographic features determined by previous studies to affect the spread of COVID‑19. ^4,8^.

1. Socioeconomic status: A measure accounting for population education, income, and occupation. Surgo Foundation developed this feature based on CDC’s Social Vulnerability Index, which accounts for population below poverty, unemployed, and without a high school diploma.
2. Household composition and disability: Developed based on CDC’s SVI, this feature accounts for populations aged 65 or older, populations aged 17 or younger, populations older than 5 years of age with a disability, and single-parent households.
3. Minority status and language: Also based on CDC’s SVI, this feature accounts for minority and populations who speak English “less than well.”
4. Housing type and transportation: Based on CDC’s SVI, this feature accounts for the population’s housing types, such as multi-unit structures, mobile homes, and crowded housing. It also accounts for populations without vehicles and those who live in group quarters.
5. Epidemiologic factors: Developed by Surgo Foundation in response to COVID‑19, this feature accounts for populations with underlying conditions (e.g., cardiovascular, respiratory, immunocompromised, obesity, and diabetes) that are vulnerable to COVID-19.
6. Healthcare system factors: Developed by Surgo Foundation for COVID-19, this factor accounts for poor health system capacity, strength, and preparedness.
7. Overall COVID-19 community vulnerability index (CCVI): This feature combines the above six features with equal weights. CCVID is a composite score that reflects the extent of a county’s vulnerability to COVID-19.

***Features related to population activities***

**Points-of-interest visits**

We used SafeGraph data, Weekly Pattern Version 2 ^9^, to calculate the number of visits to points of interest (POIs) in each county, such as restaurants, museums, hospitals, and colleges. Furthermore, to remove the influence of disparate numbers of POIs in each county, we used the percentage change based on baseline POI visits of the first week, the week of March 3, 2020.

**Social distancing index**

We used Social Distancing Metrics developed by SafeGraph ^10^, to calculate the social distancing index (SDI) of each county. The SDI was calculated dividing the number of cell phones at home by the total number of devices within a county. Also, we used the percentage change based on SDI of the first week to remove the potential influence of disparate amounts of devices in each county.

**Urban activity index**

We used Mapbox data to calculate the urban activity index. Mapbox data provides contact activity metrics in pre-defined tiles (measuring about 100 by 100 meters square) in 4-hour temporal resolution. We classified tiles into four categories and calculated an aggregated contact activity metric in classified tiles using Equation 1 to assess four kinds of urban activities on a larger scale ^11^. Therefore, urban activity index includes four sub-features: social activity index, traffic activity index, work activity index, and home activity index.

1. Social tiles: We classified tiles as social tiles in areas where at least one POI in SafeGraph is located.
2. Traffic tiles: Traffic tiles includes tiles incorporating roads.
3. Home tiles: Home tiles include tiles that cover residual buildings or have device information from 7 p.m. to 3 a.m.
4. Work tiles: all other tiles.

|  | $R_{g}=\sqrt{\frac{1}{n}\times(c_{1}^{2}+c_{1}^{2}+\ldots+c_{n}^{2})}$ | (1) |
| --- | --- | --- |

where $c_{i}$ represents contact activity metric in the tile of index $i$.

**Venables distance**

We also used Mapbox data to calculate the daily Venables distance of each county ^12^ according to Equation 2, reflecting the concentration of population activities.

|  | $D_{V}(t)=\frac{\sum_{i<j} s_{i}(t)s_{j}(t)d_{ij}}{\sum_{i<j} s_{i}(t)s_{j}(t)}$ | (2) |
| --- | --- | --- |

where $s_{i}(t)$ and $s_{j}(t)$ are the daily average activity intensities in cells $i$ and $j$, respectively, and $d_{ij}$ is the distance between two cells. The resolution of cell is 4 square meters. We also calculated percentage change based on the values of the first week to remove the influence of disparate tiles and cells in each county.

***Features related to mobility within counties***

**Cuebiq county mobility index (CMI)**

We used a county mobility index provided by Cuebiq as a feature of population mobility within counties ^13^. The Cuebiq mobility index of each county is the median of aggregated movements of each user in a day in the county (Equations 3 and 4). For example, a CMI of 5 for a county represents that the median user in that county travels 10^5^ meters (100 kilometers).

|  | $Device Mobility (DM)={log}_{10}(D+1)$ | (3) |
| --- | --- | --- |
|  | $County Mobility Index \left( CMI \right)=Median(DM)$ | (4) |

where $D$ is the aggregated distance of one user in a day in the county.

**Cuebiq shelter-in-place index (SIP)**

We used shelter-in-place index (SIP) provided by Cuebiq as another feature of population mobility within counties ^13^. Cuebiq calculated the percentage of users in each county who traveled less than 330 feet as the SIP.

***Features related to mobility across counties***

**County in-degree and out-degree centrality**

We mapped networks of movement across counties based on SafeGraph data (Weekly Pattern Version 2 ^9^). We used the data of visits from census block groups (CBGs) to POIs to map network movements across counties. The nodes in the network are counties; weights of edges are visits from CBGs in one county to POIs in another county. Therefore, the mapped network accounts only for the movements due to POI visits. We calculated the in-degree and out-degree centrality of each county in the mapped network and used the percentage change with respect to the first week to remove the influence of larger counties tending to have higher in-degree and out-degree centrality.

**Colocation degree centrality**

We used Facebook county-level colocation maps to calculate the colocation degree centrality of each county ^14^. Colocation maps could represent a network in which nodes are counties and edges represent the probability of population contacts between two counties. The network is undirected, and we calculated weighted colocation degree centrality as the feature reflecting mobility across counties. Also, we calculated the percentage change of the colocation degree centrality with respect to the first week.

***Features related to disease attributes***

**Reproduction number (R_0_)**

The reproduction number (R_0_) is an attribute of infectious diseases which estimates the number of secondary cases infected by the first case ^15^. We calculated the reproduction number of COVID-19 according to Equation 5 based on a simple epidemic transmission model ^14,16^. The model assumes that one case would infect $R_{0}$ cases after a time interval $\tau$. Then $i(0)$ infected cases at the time step 0 will lead to $i\left( t \right)=i\left( 0 \right)R^{t/\tau}$ number of infected cases at time step t.

|  | $R_{0}=e^{K\tau}$ | (5) |
| --- | --- | --- |

where $K=(\ln i\left( t \right)-\ln i\left( 0 \right))/t$, and we used $i=5.1$days for COVID-19 ^17^. Furthermore, we used the percentage changes of $R_{0}$ with respect to the first week as the feature inputs in the model.

***Features related to social network structure***

**Social connectedness index (SCI)**

We used the social connectedness index to account for social network structures affecting epidemic transmissions ^18^. The county-level social connectedness index provided by Facebook and SCI for two counties is calculated according to Equation 6 ^19^.

|  | ${SCI}_{i,j}= \frac{{FB\_Connections}_{i,j}}{{FB\_Users}_{i}\times{FB\_Users}_{j}}$ | (6) |
| --- | --- | --- |

We can find from Equation 6 that SCI of counties *i* and *j* is determined based on the number of Facebook connections (i.e., friends in Facebook) between two counties divided by the number of Facebook users in two counties. SCI, therefore, reflects the strength of social connection between two counties.

We mapped a fully connected network based on the SCI. The nodes in the network are counties; edge weights are SCIs between counties. Then we calculated weighted degree centrality of each county and the feature inputted in the model.

**2. Data related to the rollout dates of county non-pharmaceutical interventions**

We mainly used data collected by Keystone (<https://github.com/Keystone-Strategy/covid19-intervention-data>) ^20^ and Johns Hopkins University (<https://github.com/JieYingWu/COVID-19_US_County-level_Summaries/tree/master/data>) ^21^.

**3. Code availability**

The source code of the paper is shared in the Github: <https://github.com/Qingchun-Li/Unraveling-the-Dynamic-Importance-of-County-level-Features-in-Trajectory-of-COVID-19>

**4. Model related supplemental information**

We fitted five random forest classifier models each week. The dependent variable for the five models were the five classifications of the weekly new confirmed CPP (cases per 100,000 population) of each county. For each week, we put counties with zero CPP in classification 0. Then, the remaining counties were evenly divided into four classifications (i.e., classifications 1 through 4), with each classification having one of total four quantiles of CPP. Figure S1 illustrates the histograms of five classifications for five random-forest models in each week.


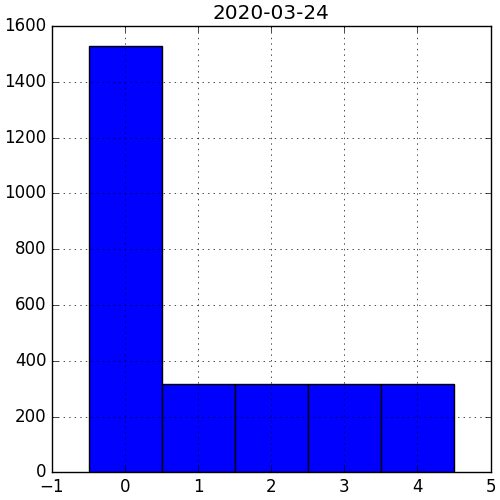

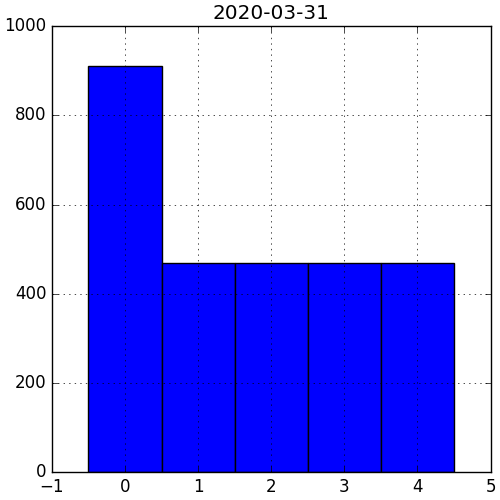

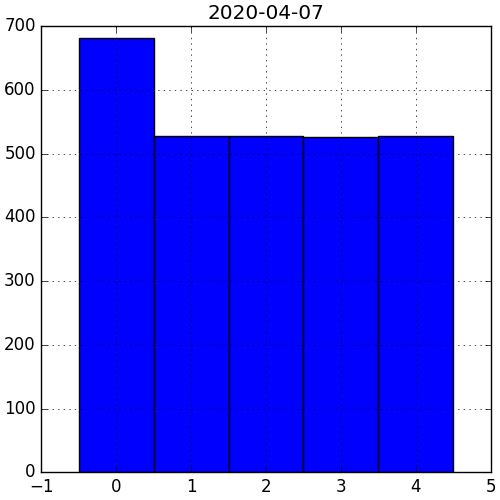

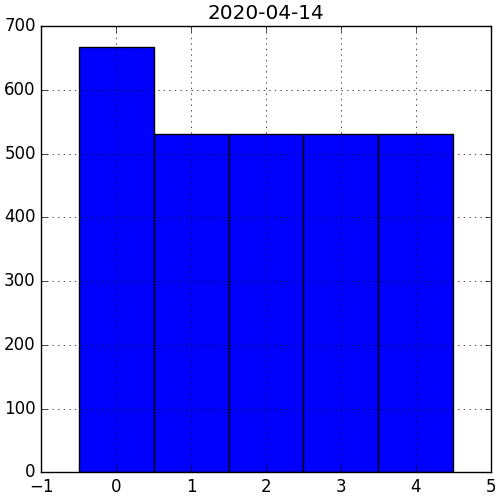

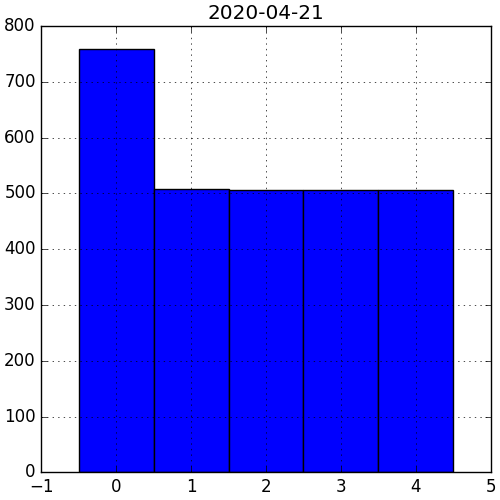

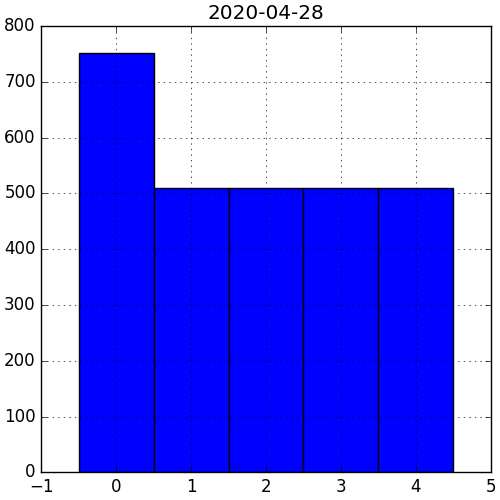


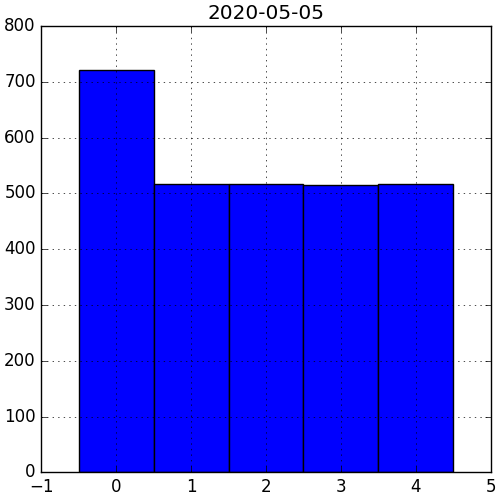

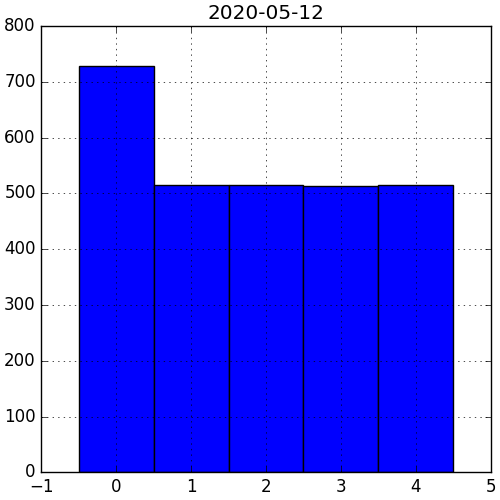

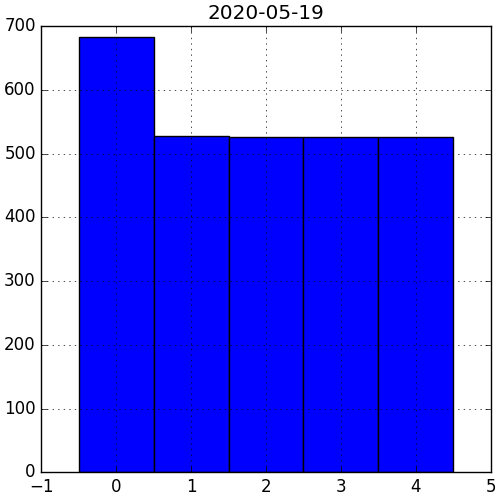


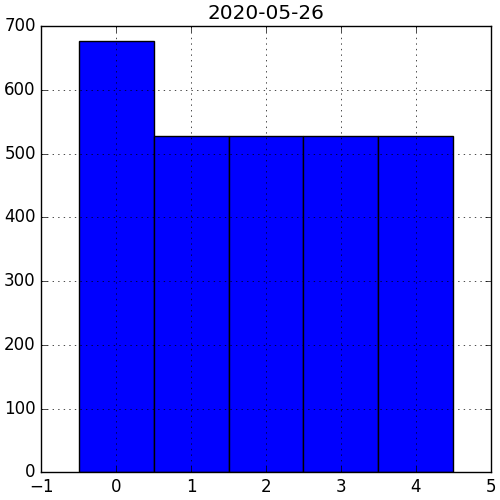

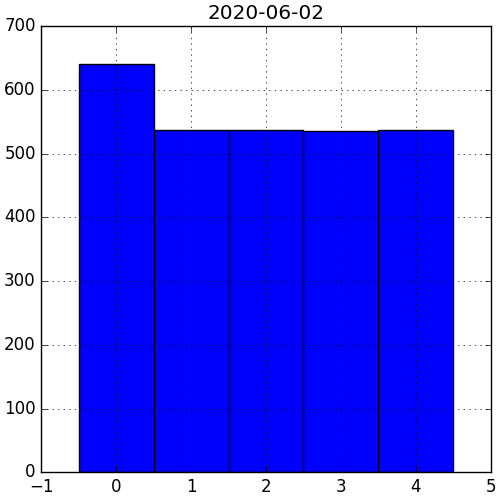

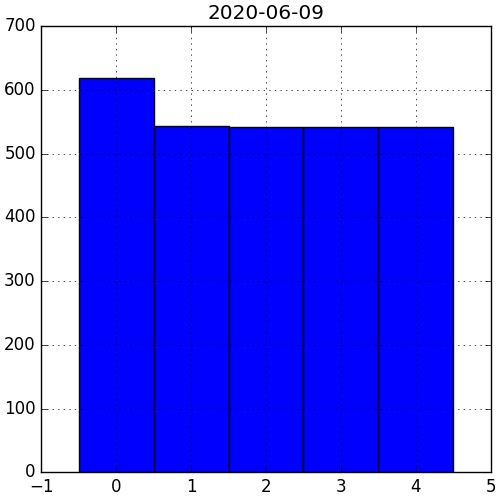


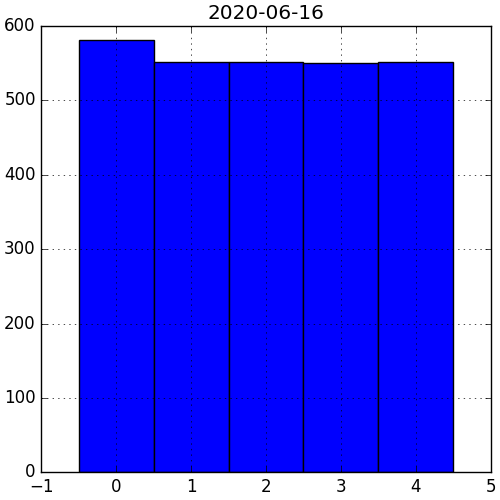

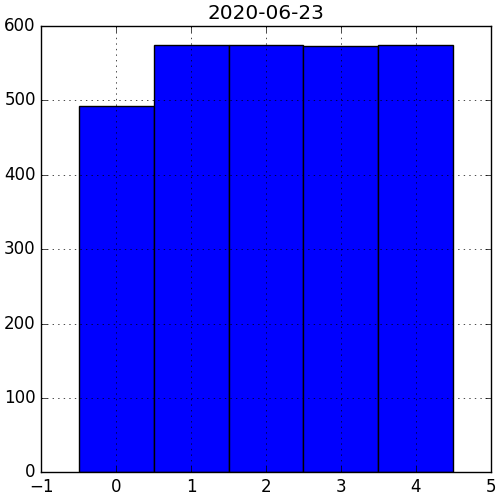


**Figure S1.** Histogram of five classifications for each week

Tables A1 to A5 shows the accuracies for the outer loop (i.e., on the test dataset) of models 1 to 5.

**Table A1 Accuracies for outer Loops (on the test dataset) in model 1.**

| Model 1 | Mean accuracy | Standard deviation | 25^th^ percentile | 75^th^ percentile |
| --- | --- | --- | --- | --- |
| 2020-03-24 | 0.624 | 0.020 | 0.610 | 0.632 |
| 2020-03-31 | 0.463 | 0.025 | 0.447 | 0.485 |
| 2020-04-07 | 0.432 | 0.025 | 0.417 | 0.449 |
| 2020-04-14 | 0.433 | 0.030 | 0.414 | 0.448 |
| 2020-04-21 | 0.452 | 0.027 | 0.435 | 0.470 |
| 2020-04-28 | 0.447 | 0.021 | 0.428 | 0.463 |
| 2020-05-05 | 0.433 | 0.023 | 0.424 | 0.452 |
| 2020-05-12 | 0.454 | 0.024 | 0.443 | 0.469 |
| 2020-05-19 | 0.443 | 0.022 | 0.424 | 0.466 |
| 2020-05-26 | 0.427 | 0.016 | 0.414 | 0.440 |
| 2020-06-02 | 0.446 | 0.025 | 0.423 | 0.460 |
| 2020-06-09 | 0.437 | 0.039 | 0.409 | 0.462 |
| 2020-06-16 | 0.436 | 0.033 | 0.413 | 0.466 |
| 2020-06-23 | 0.453 | 0.041 | 0.441 | 0.478 |

Note: model 1 includes all the counties and collected features.

**Table A2 Accuracies for outer Loops (on the test dataset) in model 2.**

| Model 2 | Mean accuracy | Standard deviation | 25^th^ percentile | 75^th^ percentile |
| --- | --- | --- | --- | --- |
| 2020-03-24 | 0.6278 | 0.018 | 0.614 | 0.638 |
| 2020-03-31 | 0.454 | 0.023 | 0.442 | 0.470 |
| 2020-04-07 | 0.426 | 0.019 | 0.410 | 0.437 |
| 2020-04-14 | 0.422 | 0.040 | 0.394 | 0.449 |
| 2020-04-21 | 0.449 | 0.016 | 0.444 | 0.456 |
| 2020-04-28 | 0.451 | 0.016 | 0.438 | 0.462 |
| 2020-05-05 | 0.424 | 0.018 | 0.413 | 0.432 |
| 2020-05-12 | 0.442 | 0.019 | 0.426 | 0.458 |
| 2020-05-19 | 0.432 | 0.025 | 0.413 | 0.455 |
| 2020-05-26 | 0.424 | 0.028 | 0.397 | 0.449 |
| 2020-06-02 | 0.437 | 0.030 | 0.420 | 0.462 |
| 2020-06-09 | 0.440 | 0.043 | 0.415 | 0.455 |
| 2020-06-16 | 0.439 | 0.030 | 0.426 | 0.463 |
| 2020-06-23 | 0.446 | 0.030 | 0.429 | 0.461 |

Note: model 2 includes all the counties and collected features excluding population density.

**Table A3 Accuracies for outer Loops (on the test dataset) in model 3.**

| Model 3 | Mean accuracy | Standard deviation | 25^th^ percentile | 75^th^ percentile |
| --- | --- | --- | --- | --- |
| 2020-03-24 | 0.398 | 0.101 | 0.330 | 0.441 |
| 2020-03-31 | 0.494 | 0.091 | 0.438 | 0.536 |
| 2020-04-07 | 0.483 | 0.101 | 0.438 | 0.527 |
| 2020-04-14 | 0.502 | 0.048 | 0.464 | 0.536 |
| 2020-04-21 | 0.498 | 0.084 | 0.438 | 0.563 |
| 2020-04-28 | 0.502 | 0.104 | 0.469 | 0.536 |
| 2020-05-05 | 0.448 | 0.090 | 0.402 | 0.518 |
| 2020-05-12 | 0.498 | 0.077 | 0.434 | 0.563 |
| 2020-05-19 | 0.473 | 0.096 | 0.406 | 0.536 |
| 2020-05-26 | 0.404 | 0.124 | 0.330 | 0.518 |
| 2020-06-02 | 0.405 | 0.094 | 0.357 | 0.455 |
| 2020-06-09 | 0.440 | 0.043 | 0.415 | 0.455 |
| 2020-06-16 | 0.420 | 0.082 | 0.357 | 0.482 |
| 2020-06-23 | 0.484 | 0.089 | 0.415 | 0.527 |

Note: model 3 includes counties with top 10% population densities and collected features excluding population density.

**Table A4 Accuracies for outer Loops (on the test dataset) in model 4.**

| Model 4 | Mean accuracy | Standard deviation | 25^th^ percentile | 75^th^ percentile |
| --- | --- | --- | --- | --- |
| 2020-03-24 | 0.381 | 0.103 | 0.321 | 0.464 |
| 2020-03-31 | 0.291 | 0.059 | 0.250 | 0.321 |
| 2020-04-07 | 0.334 | 0.057 | 0.295 | 0.357 |
| 2020-04-14 | 0.366 | 0.074 | 0.304 | 0.420 |
| 2020-04-21 | 0.376 | 0.081 | 0.312 | 0.429 |
| 2020-04-28 | 0.276 | 0.077 | 0.232 | 0.315 |
| 2020-05-05 | 0.280 | 0.061 | 0.259 | 0.321 |
| 2020-05-12 | 0.294 | 0.072 | 0.254 | 0.384 |
| 2020-05-19 | 0.284 | 0.102 | 0.214 | 0.357 |
| 2020-05-26 | 0.316 | 0.058 | 0.286 | 0.367 |
| 2020-06-02 | 0.405 | 0.073 | 0.393 | 0.455 |
| 2020-06-09 | 0.358 | 0.084 | 0.303 | 0.384 |
| 2020-06-16 | 0.430 | 0.130 | 0.321 | 0.549 |
| 2020-06-23 | 0.413 | 0.078 | 0.357 | 0.455 |

Note: model 4 includes counties with top 10%-20% population densities and collected features excluding population density.

**Table A5 Accuracies for outer Loops (on the test dataset) in model 5.**

| Model 5 | Mean accuracy | Standard deviation | 25^th^ percentile | 75^th^ percentile |
| --- | --- | --- | --- | --- |
| 2020-03-24 | 0.337 | 0.111 | 0.303 | 0.384 |
| 2020-03-31 | 0.328 | 0.058 | 0.321 | 0.357 |
| 2020-04-07 | 0.345 | 0.103 | 0.295 | 0.414 |
| 2020-04-14 | 0.277 | 0.089 | 0.223 | 0.330 |
| 2020-04-21 | 0.267 | 0.102 | 0.250 | 0.351 |
| 2020-04-28 | 0.248 | 0.088 | 0.190 | 0.313 |
| 2020-05-05 | 0.342 | 0.078 | 0.298 | 0.404 |
| 2020-05-12 | 0.281 | 0.095 | 0.193 | 0.384 |
| 2020-05-19 | 0.332 | 0.111 | 0.232 | 0.429 |
| 2020-05-26 | 0.252 | 0.080 | 0.214 | 0.294 |
| 2020-06-02 | 0.371 | 0.095 | 0.330 | 0.423 |
| 2020-06-09 | 0.324 | 0.104 | 0.226 | 0.423 |
| 2020-06-16 | 0.381 | 0.069 | 0.321 | 0.423 |
| 2020-06-23 | 0.364 | 0.078 | 0.330 | 0.414 |

Note: model 5 includes counties with top 20%-30% population densities and collected features excluding population density.

While the model accuracy on the test data is not very high, we would like to note that the objective of this paper is to investigate the relative feature importance (the rank of feature importance) in the trajectory of COVID-19 and in counties with different population densities. We do not aim to develop a predictive model with high predictive capability in this study.

**REFERENCES**

1. Rocklöv, J. & Sjödin, H. High population densities catalyse the spread of COVID-19. *Journal of travel medicine* **27**, (2020).

2. Ahmadi, M., Sharifi, A., Dorosti, S., Jafarzadeh Ghoushchi, S. & Ghanbari, N. Investigation of effective climatology parameters on COVID-19 outbreak in Iran. *Sci. Total Environ.* **729**, (2020).

3. Centers for Disease Control and Prevention. County Level Social Vulnerability Index 2018. *Centers for Disease Control and Prevention* (2020). Available at: https://data.cdc.gov/Health-Statistics/Social-Vulnerability-Index-2018-United-States-coun/48va-t53r.

4. Nepomuceno, M. R. *et al.* Besides population age structure, health and other demographic factors can contribute to understanding the COVID-19 burden. *Proceedings of the National Academy of Sciences of the United States of America* **117**, 13881–13883 (2020).

5. Sarmadi, M., Marufi, N. & Kazemi Moghaddam, V. Association of COVID-19 global distribution and environmental and demographic factors: An updated three-month study. *Environ. Res.* **188**, (2020).

6. U.S. Department of Commerce. County Level GDP. *U.S. Department of Commerce* (2020). Available at: https://apps.bea.gov/regional/downloadzip.cfm.

7. Surgo Foundation. The COVID-19 Community Vulnerability Index. *Surgo Foundation* (2020). Available at: https://precisionforcovid.org/ccvi.

8. Wright, A. L., Sonin, K., Driscoll, J. & Wilson, J. Poverty and Economic Dislocation Reduce Compliance with COVID-19 Shelter-in-Place Protocols. *SSRN Electron. J.* (2020). doi:10.2139/ssrn.3573637

9. SafeGraph. Weekly Pattern Version 2. *SafeGraph* (2020). Available at: https://docs.safegraph.com/docs/weekly-patterns.

10. SafeGraph. Social Distancing Metrics. *SafeGraph* (2020). Available at: https://docs.safegraph.com/docs/social-distancing-metrics.

11. Gao, X. *et al.* Early Indicators of Human Activity During COVID-19 Period Using Digital Trace Data of Population Activities. *Front. Built Environ.* **6**, 223 (2021).

12. Louail, T. *et al.* Uncovering the spatial structure of mobility networks. *Nat. Commun.* **6**, (2015).

13. Cuebiq. Cuebiq’s COVID-19 Mobility Insights. *Cuebiq* (2020). Available at: https://help.cuebiq.com/hc/en-us/articles/360041285051-Cuebiq-s-COVID-19-Mobility-Insights#h_4e44ff71-27e9-4b83-977e-d18911b21817.

14. Fan, C. *et al.* Effects of population co-location reduction on cross-county transmission risk of COVID-19 in the United States. *Appl. Netw. Sci.* **6**, 1–18 (2021).

15. Dietz, K. The estimation of the basic reproduction number for infectious diseases. *Stat. Methods Med. Res.* **2**, 23–41 (1993).

16. Ramchandani, A., Fan, C. & Mostafavi, A. DeepCOVIDNet: An Interpretable Deep Learning Model for Predictive Surveillance of COVID-19 Using Heterogeneous Features and Their Interactions. *IEEE Access* **8**, 159915–159930 (2020).

17. Zhang, J. *et al.* Changes in contact patterns shape the dynamics of the COVID-19 outbreak in China. *Science (80-. ).* **368**, 1481–1486 (2020).

18. Kuchler, T., Russel, D. & Stroebel, J. The geographic spread of COVID-19 correlates with structure of social networks as measured by Facebook. *arXiv e-prints: 2004.03055* (2020).

19. Facebook. Facebook Social Connectedness Index. *Facebook* (2020). Available at: https://dataforgood.fb.com/docs/social-connectedness-index-methodology/.

20. Chen, X. & Qiu, Z. Scenario Analysis of Non-Pharmaceutical Interventions on Global Covid-19 Transmissions. *arXiv* (2020).

21. Killeen, B. D. *et al.* A county-level dataset for informing the United States’ response to covid-19. *arXiv* (2020).
